# Consumer physical activity tracking device ownership and use among a population-based sample of adults

**DOI:** 10.1371/journal.pone.0189298

**Published:** 2018-01-02

**Authors:** Soultana Macridis, Nora Johnston, Steven Johnson, Jeff K. Vallance

**Affiliations:** 1 Alberta Centre for Active Living, Edmonton, Alberta, Canada; 2 Faculty of Physical Education and Recreation, University of Alberta, Edmonton, Canada; 3 Faculty of Health Disciplines, Athabasca University, Athabasca, Canada; University of Illinois at Urbana-Champaign, UNITED STATES

## Abstract

Consumer physical activity tracking devices (PATs) have gained popularity to support individuals to be more active and less sedentary throughout the day. Wearable PATs provide real-time feedback of various fitness-related metrics such as tracking steps, sedentary time, and distance walked. The purpose of this study was to examine the prevalence and correlates of PAT ownership and use among a population-based sample of adults. A representative sample of adults ≥18 years (N = 1,215) from Alberta, Canada were recruited through random-digit dialing and responded to a questionnaire via computer-assisted telephone interviewing methods in summer 2016. Questionnaires assessed demographic and health behaviour variables, and items were designed to assess PAT ownership and usage. Logistic regression analysis (odds ratios) was used to assess correlates of PAT ownership and use. On average, participants (N = 1,215) were 53.9 (SD 16.7) years and 50.1% were female. Of the sample, 19.6% (n = 238) indicated they currently own and use a PAT. Participants who owned a PAT wore their device on average 23.2 days within the past month. Currently owning a PAT was significantly associated with being female (OR = 1.41, CI: 1.10 to 1.82), being <60 years of age (OR = 1.86, CI: 1.37 to 2.53), having at least some post secondary education (OR = 1.88, CI: 1.36 to 2.60), having a BMI ≥25 (OR = 1.52, CI: 1.16 to 1.99), and meeting physical activity guidelines (OR = 1.45, CI: 1.12 to 1.88). Similar correlates emerged for PAT use. Correlates significantly associated with PAT use and ownership included being female, being less than 60 years of age, having a post-secondary education, meeting physical activity guidelines, and being overweight/obese. This is the first study to examine characteristics of PAT ownership and use among Canadian adults.

## Introduction

Physical inactivity and sedentary behaviour are risk factors for many chronic physical and mental health conditions, such as pre-mature mortality, cardiovascular disease, stroke, hypertension, colon cancers, depression, anxiety, breast cancer and diabetes [1, 2]. Although it is recommended that adults accumulate up to 150 minutes of moderate-to-vigorous physical activity per week [3] which habitually, has shown to have a protective effect against chronic diseases, [4–6] the overwhelming majority of adults do not meet physical activity guidelines [7, 8].

Consumer physical activity tracking devices (PATs) are one tool that may support individuals to be more active and less sedentary throughout the day. A PAT is a device that is worn that monitors and tracks physical activity and/or sedentary-related metrics, such as number of steps, overall physical activity, sedentary time, distance walked or ran, and in some cases, heart rate, flights of stairs, calories and sleep patterns. Examples of activity tracking devices include wearable devices (e.g. Fitbit, Garmin, Jawbone), smart watches (e.g. Apple Watch, Galaxy Gear, Samsung Gear), phone applications (e.g. Apple Health, Samsung Health, Google Fit), and step pedometers [9].

Behavioural physical activity interventions incorporating a PAT have increased physical activity among overweight or obese adults [10]. Use of a pedometer has been found to be significantly associated with increases in physical activity and decreases in BMI and blood pressure [11]. More specifically, physical activity interventions using pedometers with adults found a 26.9% increase in physical activity levels compared to baseline activity level [11]. Smartphone applications have shown to support weight loss interventions [12, 13] and increases in physical activity [14]. PATs have been documented to show promise in clinical populations [15–18]. For example, the continuous monitoring that such devices provide may have implications for enhancing the care and recovery of hospitalized patients by recording relevant patient health metrics and physical activity, which can help to facilitate “remote, real-time monitoring of medical conditions, enable disease management, and provide patient education [19]. Some PATs incorporate behaviour change techniques, such as self-monitoring, goal-setting, feedback, prompts/cues and rewards, to support and motivate individuals to achieve activity goals [9, 20]. The continuous real-time feedback of activity trackers provides an opportunity for practitioners to explore strategies to develop and implement large-scale and low-cost physical activity and sedentary behaviour interventions.

Given the increasing visibility and use of PATs, research is just starting to emerge regarding ownership and use of PATs among adults. The purpose of this study was to examine the prevalence and correlates of PAT ownership and use among a population-based sample of adults. The secondary purpose was to determine the most commonly used PATs and the types of functions that were considered to be useful among those who currently own and use a PAT.

## Methods

### Participants and design

This study was approved by the Research Ethics Board at the University of Alberta. All participant recruitment and data collection were conducted through a centralized research unit—the Population Research Laboratory (PRL) at the University of Alberta (Edmonton, Alberta, Canada). The PRL specializes in the gathering, analysis, and presentation of data about demographic, social and public issues and has particular expertise in administering computer-assisted telephone interviewing (CATI) surveys. Representative random sampling was conducted through random-digit dialing telephone interviews through landlines and cellphones between June and August, 2016. Participants were 18 years of age or older and were living in a household that could be contacted by direct dialing. All participants were informed about the details and purpose of this voluntary questionnaire, and were provided an opportunity to provide verbal consent. A sampling aim of 1,200 households across Alberta, with a minimum of 400 respondents each in Metropolitan Edmonton, Metropolitan Calgary, and from the rest of the province. Participants targeted were 18 years of age or older. The sample size obtained was 1,215.

### Measures

All measures were self-reported via telephone interview. Sociodemographic variables included gender, age, education, employment status, marital status, height, and weight (subsequently used to calculate BMI (kg/m^2^), which was dichotomized into normal versus overweight/obese), and region (see Table 1).

**Table 1:** Characteristics of respondents (N = 1215).

Characteristic	N (%)	Mean (SD)
**Age**		
18 to 59 years	726 (59.8)	53.9 years (16.7)
≥ 60 years	489 (40.2)	
**Gender**		
Male	606 (49.9)	
Female	609 (50.1)	
**Marital status**		
Not married	441 (36.3)	
Married/Common-law	766 (63.0)	
**Education**		
≤ High school	294 (24.2)	
Pursued post-secondary education	914 (75.2)	
Employment*		
Not working	582 (47.9)	
Working full or part time	630 (51.9)	
**Region**		
Metro Edmonton / Calgary	807 (66.4)	
Other Alberta	408 (33.6)	
**BMI**		27.3 kg/m^2^ (5.9)
Overweight/Obese	727 (59.8)	
Normal weight	441 (43.2)	
**Sedentary Behaviour–Weekday**		9 hrs (4.7)
< 9 hours	525 (43.2)	
≥ 9 hours	690 (56.8)	
**Sedentary Behaviour–Weekend**		8.5 hrs (4.8)
< 9 hours	452 (37.2)	
≥ 9 hours	763 (62.8)	
**Leisure-Time Physical Activity**		43.7 MET-mins/week (36.2)
Insufficiently active	575 (47.3)	
Sufficiently active	640 (52.7)	

*Not working includes individuals who are retired, unemployed, on maternity leave, are students, or are on disability.

Leisure-time physical activity (LTPA) was assessed using the Godin Shephard Leisure-Time Exercise Questionnaire [21]. Participants were asked to indicate how many times in an average week they engaged in mild, moderate, and strenuous exercise for more than 15 minutes. Weekly LTPA was calculated as the sum of weighted minutes of mild, moderate and strenuous activity by associated metabolic equivalent value of a task (MET) of 3, 5, and 9, respectively [22, 23]. Physical activity intensity is often expressed in METs—a measure of energy output equal to one’s basal resting metabolic rate, which is assumed to be 3.5 mL•kg-1•min-1 [24]. Participants were considered sufficiently active based on an MET score of ≥38 per week for men and ≥35 per week for women [25].

The Sedentary Behaviour Questionnaire [26] was used to estimate time spent in sedentary behavior during a typical week. Nine types of sedentary activities were assessed including: watching television, playing computer/video games, sitting while listening to music, sitting and talking on the phone, doing paperwork or office work, sitting and reading, playing a musical instrument, doing arts and crafts, and sitting to drive or ride in a car, bus or train. Scores were summed to calculate sedentary time in minutes per weekdays and weekend days. To obtain weekly estimates, weekday minutes were multiplied by 5 and weekend minutes were multiplied by 2 and these were summed for total hours/week. Average sedentary behaviour time per week was calculated by summing weekday and weekend sedentary behaviour then dividing by seven days for the week.

Participants were asked a set of questions related to wearable PATs. To determine ownership and use, participants were asked, “Do you currently use an activity tracker for the purposes of tracking how active you are throughout the day?” and were provided the following answer options, “Yes, I use an activity tracker,” “No, I own an activity tracker but do not use it,” and “I do not own an activity tracker.” Of those who owned a PAT, participants were asked to indicate the type of PAT that they use (open-ended), and of those who did not own a PAT, participants were asked if they had plans to use one in the future (yes/no/not sure). Those participants who owned or planned to own a device were asked to indicate the type of activity tracking functions they consider or would consider to be useful, somewhat useful, or very useful. Functions included a) tracking types of activities (e.g., walking, cycling), b) steps, c) flights of stairs climbed, d) distance travelled, e) duration of activities, f) sedentary time, g) sleep time, h) calories burned, i) heart rate, j) GPS (Global Positioning System), k) inactivity/sedentary alert, and l) connecting with friends/family for activity challenges.

Finally, to understand frequency of PAT use, participants who owned and currently used a device were asked, “Thinking about a typical month, how many days on average do you use your activity tracker?” Of those who owned and no longer used their device, these participants were asked, “If you are not currently using your activity tracker, how long did you use it before you stopped using it?” Both questions were answered in number of days.

### Analysis

Statistical analyses were performed using SPSS version 24. Prior to any analyses, data were checked (via frequency distributions) for outliers and discrepancies. Frequency counts and percentages were also calculated to determine the PAT ownership and related characteristics. Logistic regression (i.e., odds ratios) was used to determine associations of demographic and health-related characteristics with PAT variables. Odds ratios (ORs) reflect the increase (or decrease if the ratio is <1) in odds of being in one outcome category when the value of the predictor increases by one unit. In all analyses, adjusted ORs, as well as the associated 95% confidence interval (CI), are presented for each level of the variable in comparison with the lowest (referent) level. The population-based dataset that was used for this study had a limited number of sociodemographic and clinical variables, therefore, common predictor variables were entered into each model. These predictors included gender (male v. female), age (60 years of age or below), education (at least college/university education), marital status (married/common-law v. not married/common-law), employment status (working full or part time v. not working), rural (rural v. urban residence), BMI (normal v. overweight/obese), average weekday sedentary time (above 9 hrs/day v. below 9 hrs/day), average weekend sedentary time (above 9 hrs/day v. below 9 hrs/day), and physical activity (meeting physical activity guidelines v. not meeting). The same set of predictor variables were entered into each model. A full model approach (without any variable selection to a reduced model) was used.

## Results

Of households contacted, 21% responded to the survey. The random sample of 1,215 is considered accurate within +/- 2.8% (CI: 95%), with subsamples of 400 considered accurate within +/-5.0% (CI: 95%). On average, it took two call attempts to complete an interview and 98% of completed interviews were completed with five attempts. Further information depicting the number of call attempts made to complete an interview can be found in supporting information files (S1 Table).

Table 2 shows the sample characteristics of respondents. On average, participants were 53.9 (SD = 16.7; range = 75) years of age with a mean BMI of 27.3 kg/m^2^ (SD = 5.9; range = 47.1), and half were female. The majority of participants had pursued a post-secondary education (75.2%) and 63.0% were married or in a common-law relationship. Participants were found to spend an average of 9 hours (SD = 4.7) and 8.5 (SD = 4.8) hours in sedentary behaviour on weekdays and weekends, respectively. Slightly over half (52.7%) of participants achieved sufficient LTPA (i.e., MET score of ≥38 per week for men and ≥35 per week for women). Table 2 provides further breakdown of sample characteristics into three categories: i) % do not use, ii) % own, but do not use, and iii) % own, and use.

**Table 2:** Percentage of ownership and use across demographic and health characteristics (N = 1215).

Characteristic	Do not own N (%)	Own, but do not use N (%)	Own, and use N (%)	P value*
**Age**				
18 to 59 years	556 (46%)	168 (13.9%)	219 (18.1%)	< .001
≥ 60 years	193 (16%)	35 (2.9%)	37 (3.1%)	
**Gender**				
Male	403 (33.4%)	87 (7.2%)	118 (9.8%)	.007
Female	346 (28.6%)	116 (9.6%)	138 (11.4%)	
**Marital status**				
Not married	316 (26.3%)	83 (6.9%)	86 (7.2%)	.048
Married/Common-law	429 (35.8%)	117 (9.8%)	169 (14.1%)	
**Education**				
≤ High school	207 (17.2%)	42 (3.5%)	35 (2.9%)	< .001
Pursued post-secondary education	538 (44.7%)	161 (13.4%)	220 (18.3%)	
**Employment**				
Not working	295 (24.5%)	73 (6.1%)	82 (6.8%)	.091
Working full or part time	451 (37.4%)	130 (10.8%)	174 (14.4%)	
**Region**				
Metro Edmonton / Calgary	497 (60.5%)	129 (10.7%)	195 (68%)	.005
Other Alberta	252 (20.9%)	74 (6.1%)	387 (32%)	
**BMI**				
Overweight/Obese	407 (35.1%)	106 (9.1%)	162 (14%)	.031
Normal weight	312 (26.9%)	88 (7.6%)	86 (7.4%)	
**Sedentary Behaviour–Wk/day**				
< 9 hours	344 (28.5%)	97 (8%)	113 (9.4%)	.738
≥ 9 hours	405 (33.5%)	106 (8.8%)	143 (11.8%)	
**Sedentary Behaviour–Wk/end**				
< 9 hours	313 (25.9%)	78 (6.5%)	90 (7.4%)	.162
≥ 9 hours	437 (36.1%)	125 (10.3%)	166 (13.7%)	
**Leisure-Time Physical Activity**				
Insufficiently active	339 (28.1%)	88 (7.3%)	91 (7.5%)	.025
Sufficiently active	410 (33.9%)	115 (9.5%)	165 (13.7%)	

* Chi square

A total of 35.5% of the sample own a PAT, however only 19.6% currently use their device. FitBit^®^ was the most commonly reported brand (32.5%). Of those who do not own or do not use their PAT, 13.8% indicated that they plan to use one in the future, and 3.8% were unsure. Participants who own and used their PAT wore their device an average of 23.2 (SD = 9.7) days within the past month. Of the 254 participants who indicated at least one day of use in a typical month, 51% indicated they wore their PAT on at least 30 days in a typical month. Of those participants who own a PAT, but no longer use their device (15.9%), the average number of days worn before stopping use was 240.2 days (SD = 383.3), or approximately 8 months. Participants who currently own or plan to use a PAT in the future indicated that distance travelled (89.4%), types of activity (88.4%), and tracking steps (89.1%) were the most useful functions. Functions that were considered not useful were connecting with friends/family for step challenges (50.9%), Global Position System (GPS; 44.1%), and inactivity/sedentary alerts (43.4%).

Logistic regression analyses revealed that currently *owning* a PAT was significantly associated with being female (OR = 1.41, CI: 1.10 to 1.82), being <60 years of age (OR = 1.86, CI: 1.37 to 2.53), having at least some post secondary education (OR = 1.88, CI: 1.36 to 2.60), having a BMI ≥25 (OR = 1.52, CI: 1.16 to 1.99), and meeting physical activity guidelines (OR = 1.45, CI: 1.12 to 1.88). No significant associations were found for region and sedentary time.

Currently *using* a PAT was significantly associated with being female (OR = 1.56, CI: 1.15 to 2.11), being <60 years of age (OR = 1.75, CI: 1.21 to 2.54), having at least some post secondary education (OR = 1.88, CI: 1.23 to 2.85), being married (OR = 1.42, CI: 1.02 to 1.99), having a BMI ≥25 (OR = 1.85, CI: 1.33 to 2.57), living in an urban area (OR = 1.47, CI: 1.05 to 2.06), and meeting physical activity guidelines (OR = 1.70, CI: 1.24 to 2.32). No significant associations were found for sedentary time.

Logistic regression was also used to analyze correlates of non-use (i.e., own a PAT, but currently do not use it). For this analysis, the sample size was N = 408 (n = 246: currently use their PAT; n = 192; currently own but do not use their PAT). Results indicated participants who were overweight/obese were more likely to not be currently using their PAT (OR = 1.75, CI: 1.14 to 2.68) and people who were meeting physical activity guidelines were significantly less likely to be currently using their PAT (OR = .64., CI: .43 to .96).

## Discussion

The purpose of this study was to examine the prevalence and correlates of PAT ownership and use among a population-based sample of adults. The overall findings suggest that many people in the province of Alberta, Canada are interested in and/or own a PAT. Our sample was a broad representation of the province, with samples equally distributed among the two largest metropolitan areas, Edmonton and Calgary, as well as the rest of the province outside of these larger metropolitan areas. Overall, 19.6% (n = 238) indicated they currently own and use a PAT, with FitBit^®^ as the most commonly reported brand (32.5%). Correlates significantly associated with PAT use and ownership included being female, being less than 60 years of age, having a post-secondary education, meeting physical activity guidelines, and being overweight/obese. To our knowledge, this is the first study to examine characteristics of consumer wearable ownership and use among Canadian adults.

Of the sample, nearly one-third owned a PAT, yet only one-fifth currently used their device. Of those who did not own a PAT, just over 10% were planning to use one in the future, 3.8% were not sure, and the remainder indicated no plans to use a PAT in the future. Similar findings were observed in a cross-sectional national telephone survey conducted in Australia with 1,349 participants, whereby over one-third had used a PAT, 16% were interested in using a PAT, 5% were unsure, and 44% were not interested [27]. Our study found that participants used their device for an average of eight months before stopping. Comparatively, Alley and colleagues [27] found that of those who used a PAT, 37% used it or less than a month, 35% used it between one and six months, and 27% used it for more than six months. Although not examined in our study, previous research has identified barriers to PAT use and include: not wanting to increase their physical activity, do not think a PAT would be helpful, high cost, technology, lack of time, being too old, health, or no interest [27]. Our study suggests a significant portion of the population owns a PAT however, PAT use is often short term or intermittent.

There are a wide variety of PATs available to consumers for personal use. FitBit^®^ was the most commonly reported device with approximately one-third of owners using this brand. Alley and colleagues [27] reported pedometers were most commonly used followed by advanced PATs such as heart rate monitors, accelerometers (such as FitBit^®^), and smartphone applications. Between our studies, there may be differences in popularity as pedometers have been available for a longer time, are simple to use, and have a minimal cost. However, in recent years, PAT technology has become more popular, accessible, and appealing with a variety of designs, brands, and functions. Further, their data may be reflective of the time and context in which the data were collected (i.e., 2014).

The PATs most useful functions identified were tracking distance travelled, types of activity, and tracking steps. The three least useful functions identified were connecting with friends/family for activity challenges, GPS, and inactivity/sedentary alerts. The popularity of tracking steps may be due to the fact that step counting can be easy to comprehend, measure and interpret, and can be motivational towards increase physical activity [11]. Similar findings were found among older adults in a 12-week pilot study examining feasibility and utility of wristband PATs [27]. Aside from PATs being easy to use, participants liked that the PAT made them aware of their daily diet and movements, as well as daily steps and motivation to reach or exceed step goals [28]. Similarities found between our study and the literature provide insight on popular functions, which can inform researchers and public health practitioners on how to engage and challenge adults to be more active and less sedentary throughout the day.

Currently owning and using a PAT was significantly associated with being female, being less than 60 years of age, having a least some post-secondary education, living in a metropolitan area (PAT use only), having a BMI greater or equal to 25, and meeting physical activity guidelines. Similar findings related to gender and level of education were found in studies with pedometers [27, 29]. However, two of these studies also found that use of pedometers was more likely among middle-aged adults (44–64 years) [29, 30]. It has been suggested that those who are least interested in increasing their physical activity levels and lead sedentary lifestyles tend to be inactive, males, and those with less education [31, 32]. Yet, interventions utilizing a PAT have been found to be most effective increasing daily physical activity among those individuals who are inactive compared to those who are active.[10, 11, 33–35] At the same time, research has found those living in rural areas tend to be less active and more sedentary than those living in urban areas [36, 37].

Data from this study may be useful for those developing PAT-based interventions, and larger public health initiatives that can reach a larger population at a low cost. UWALK (www.uwalk.ca) is one example of a multi-strategy, community-wide, e- and m-Health physical activity promotion program in Alberta, Canada. Building upon the success of the 10,000 Steps program in Australia, [38, 39] UWALK promotes physical activity through the accumulation of steps and stairs, and allows individuals to manually enter, or synchronize their PATs, to self-monitor daily activity on a private account. Individual members are also able to engage with other individuals, teams and/or or communities, and develop or participate in interactive challenges.[40] Over three years, UWALK accumulated 16,061 registered members who were mostly female (79%) with an average age of 45 (SD 13), and accumulated 5,230,677,746 steps. The capabilities of PATs combined with community-wide interventions, such as UWALK, provide opportunities for wider reach of physical activity and sedentary interventions and data collection to monitor and evaluate real-time activity of a sample or population group. PATs can also be utilized to motivate people to develop and achieve physical activity goals, which in turn can support chronic disease management and prevention that have been linked to insufficient physical activity levels and excessive sedentary behaviour time [35, 41–43]. Yet, there is still a need to understand delayed effects of intervention prompts, as the suggestion may not result in immediate engagement in the activity. Rather, it may motivate the individual to be more active over the course of their day [44].

A strength of our study is the recruitment of a population-based sample. This study also provides indication that many adults are interested in PATs, and reveal the device functions that are most appealing to participants. Furthermore, correlates of PAT use among this population sample were identified, which can support researchers and practitioners seeking to develop interventions with PATs, as we outline above. The cross-sectional self-reported nature of the survey may have introduced some recall bias among participants. Due to rapid changes in technology for devices and smartphone applications, future findings may evolve. There is a need for future research to explore barriers and facilitators of PAT ownership and use, as well as preferred functions and features that are useful, engaging, and promote long-term use. As highlighted by Dempsey and colleagues [44], rigorous statistical approaches for understanding behavioural aspects in relation to PATs has lagged behind the advancements of the technology. However, micro-randomised trials–participants randomly assigned a treatment from a set of possible treatments several times a day–may provide a first step towards designing studies in this field. In turn, the approach may help in the development and optimization of real-time mobile health interventions [44].

Based on the high prevalence of PAT ownership and interest identified in our study, PATs may be a promising tool for researchers and public health practitioners to engage people in physical activity and support reductions in sedentary behaviours. Our study findings also provide insight on factors to consider when tailoring physical activity and sedentary behaviour interventions for various population groups in terms of demographics, but also in terms of PAT function preferences. There may also be implications of use based on whether individuals live in urban or rural environments that should be considered. PATs provide an opportunity to reach a wider audience and may be useful for support implementation and evaluation of population-level interventions.

## Supporting information

S1 Table Number of completions by call attempts. (DOCX)

## References

[1] Owen N, Healy GN, Matthews CE, Dunstan DW. Too much sitting: the population health science of sedentary behavior. Exerc Sport Sci Rev. 2010;38(3):105–13. doi: 10.1097/JES.0b013e3181e373a2 20577058 10.1097/JES.0b013e3181e373a2 PMC3404815

[2] Warburton DER, Nicol CW, Bredin SSD. Health benefits of physical activity: the evidence. Can Med Assoc J. 2006;174:801–9. 16534088 10.1503/cmaj.051351 PMC1402378

[3] Biswas A, Oh PI, Faulkner GE, Bajaj RR, Silver MA, Mitchell MS, et al Sedentary time and its association with risk for disease incidence, mortality, and hospitalization in adults: a systematic review and meta-analysis. Ann Int Med. 2015;162(2):123–32. doi: 10.7326/M14-1651 25599350 10.7326/M14-1651

[4] Warburton DER, Katzmarzyk PT, Rhodes RE, Shephard RJ. Evidence-informed physical activity guidelines for Canadian adults. Appl Phys Nutr Metab. 2007;32(S2E):S16–S68. 18213940

[5] Warburton DE, Nicol CW, Bredin SS. Health benefits of physical activity: the evidence. Can Med Ass J. 2006;174(6):801–9. 16534088 10.1503/cmaj.051351 PMC1402378

[6] Lee IM. Relative intensity of physical activity and risk of coronary heart disease. Circulation. 2003;107(8):1110–6. 12615787 10.1161/01.cir.0000052626.63602.58

[7] Colley RC, Garriguet D, Janssen I, Craig CL, Clarke J, Tremblay MS. Physical activity of Canadian adults: accelerometer results from the 2007 to 2009 Canadian Health Measures Survey. Health Reports. 2011;22(1):7 21510585

[8] Hallal PC, Andersen LB, Bull FC, Guthold R, Haskell W, Ekelund U, et al Global physical activity levels: surveillance progress, pitfalls, and prospects. The Lancet. 2012;380(9838):247–57. 10.1016/S0140-6736(12)60646-1 22818937

[9] Mercer K, Li M, Giangregorio L, Burns C, Grindrod K. Behavior change techniques present in wearable activity trackers: a critical analysis. JMIR Mhealth Uhealth. 2016;4(2):e40 doi: 10.2196/mhealth.4461 27122452 10.2196/mhealth.4461 PMC4917727

[10] de Vries HJ, Kooiman TJ, van Ittersum MW, van Brussel M, de Groot M. Do activity monitors increase physical activity in adults with overweight or obesity? A systematic review and meta-analysis. Obesity. 2016;24(10):2078–91. doi: 10.1002/oby.21619 27670401 10.1002/oby.21619

[11] Bravata DM, Smith-Spangler C, Sundaram V, Gienger AL, Lin N, Lewis R, et al Using pedometers to increase physical activity and improve health: a systematic review. JAMA. 2007;298(19):2296–304. doi: 10.1001/jama.298.19.2296 18029834 10.1001/jama.298.19.2296

[12] Carter MC, Burley VJ, Nykjaer C, Cade JE. Adherence to a smartphone application for weight loss compared to website and paper diary: pilot randomized controlled trial. J Med Internet Res. 2013;15(4):e32 doi: 10.2196/jmir.2283 23587561 10.2196/jmir.2283 PMC3636323

[13] Allen JK, Stephens J, Dennison Himmelfarb CR, Stewart KJ, Hauck S. Randomized controlled pilot study testing use of smartphone technology for obesity treatment. J Obes. 2013;2013:151597 doi: 10.1155/2013/151597 24392223 10.1155/2013/151597 PMC3872411

[14] Glynn LG, Hayes PS, Casey M, Glynn F, Alvarez-Iglesias A, Newell J, et al Effectiveness of a smartphone application to promote physical activity in primary care: the SMART MOVE randomised controlled trial. Br J Gen Pract. 2014;64(624):e384–91. doi: 10.3399/bjgp14X680461 24982490 10.3399/bjgp14X680461 PMC4073723

[15] Vallance JKH, Courneya KS, Plotnikoff RC, Yasui Y, Mackey JR. Randomized controlled trial of the effects of print materials and step pedometers on physical activity and quality of life in breast cancer survivors. J Clin Onc. 2007;25(17):2352–9. 10.1200/JCO.2006.07.9988 17557948

[16] Culhane KM, Lyons GM, Hilton D, Grace PA, Lyons D. Long-term mobility monitoring of older adults using accelerometers in a clinical environment. Clin Rehabil. 2004;18:335–4. doi: 10.1191/0269215504cr734oa 15137565 10.1191/0269215504cr734oa

[17] Dalton A, Khalil H, Busse M, Rosser A, van Deursen R, Olaighin G. Analysis of gait and balance through a single triaxial accelerometer in presymptomatic and symptomatic Huntington's disease. Gait Posture. 2013;37(1):49–54. doi: 10.1016/j.gaitpost.2012.05.028 22819009 10.1016/j.gaitpost.2012.05.028

[18] Kuo YL, Culhane KM, Thomason P, Tirosh O, Baker R. Measuring distance walked and step count in children with cerebral palsy: an evaluation of two portable activity monitors. Gait Posture. 2009;29(2):304–10. doi: 10.1016/j.gaitpost.2008.09.014 19019680 10.1016/j.gaitpost.2008.09.014

[19] Appelboom G, Yang AH, Christophe BR, Bruce EM, Slomian J, Bruyere O, et al The promise of wearable activity sensors to define patient recovery. J Clin Neurosci. 2014;21(7):1089–93. doi: 10.1016/j.jocn.2013.12.003 24534628 10.1016/j.jocn.2013.12.003

[20] Lyons EJ, Lewis ZH, Mayrsohn BG, Rowland JL. Behavior change techniques implemented in electronic lifestyle activity monitors: a systematic content analysis. J Med Internet Res. 2014;16(8):e192 doi: 10.2196/jmir.3469 25131661 10.2196/jmir.3469 PMC4147713

[21] Godin G, Shephard RJ. A simple method to assess exercise behavior in the community. Can J Appl Sport Sci. 1985;10(3):141–6. 4053261

[22] Healthy Lifestyles Research Center—Arizona State University. Compendium of Physical Activities: Activity Categories 2011 [15 March 2017]. Available from: https://sites.google.com/site/compendiumofphysicalactivities/home.

[23] Ainsworth BE, Haskell WL, Herrmann SD, Meckes N, Bassett DR Jr, Tudor-Locke C, et al 2011 Compendium of Physical Activities: a second update of codes and MET values. Medicine and science in sports and exercise. 2011;43(8):1575–81. doi: 10.1249/MSS.0b013e31821ece12 21681120 10.1249/MSS.0b013e31821ece12

[24] Tremblay MS, Shephard RJ, Brawley LR. Research that informs Canada’s physical activity guides: an introduction. Appl Physiol Nutr Metab. 2007;32(S2E):S1–S8. 18213938

[25] Garcia Bengoechea E, Spence JC, McGannon KR. Gender differences in perceived environmental correlates of physical activity. Int J Beh Nutr Phys Act. 2005;2:12. 10.1186/1479-5868-2-12 PMC1253529 16159404

[26] Rosenberg DE, Norman GJ, Wagner N, Patrick K, Calfas KJ, Sallis JF. Reliability and validity of the Sedentary Behavior Questionnaire (SBQ) for adults. J Phys Act Health. 2010;7(6):697–705. 21088299 10.1123/jpah.7.6.697

[27] Alley S, Schoeppe S, Guertler D, Jennings C, Duncan MJ, Vandelanotte C. Interest and preferences for using advanced physical activity tracking devices: results of a national cross-sectional survey. BMJ Open. 2016;6(7):e011243 doi: 10.1136/bmjopen-2016-011243 27388359 10.1136/bmjopen-2016-011243 PMC4947799

[28] O'Brien T, Troutman-Jordan M, Hathaway D, Armstrong S, Moore M. Acceptability of wristband activity trackers among community dwelling older adults. Geriatr Nurs. 2015;36(2 Suppl):S21–5. 25771957 10.1016/j.gerinurse.2015.02.019

[29] Eakin EG, Mummery K, Reeves MM, Lawler SP, Schofield G, Marshall AJ, et al Correlates of pedometer use: results from a community-based physical activity intervention trial (10,000 Steps Rockhampton). Int J Beh Nutr Phys Act. 2007;4:31. 10.1186/1479-5868-4-31 PMC1950707 17655770

[30] Craig CL, Cragg SE, Tudor-Locke C, Bauman A. Proximal impact of Canada on the move: the relationship of campaign awareness to pedometer ownership and use. Can J Pub Health. 2006;97 (Suppl 1):S21–S7. 10.1007/BF03405361 PMC6975740 16676835

[31] Caperchione CM, Vandelanotte C, Kolt GS, Duncan M, Ellison M, George E, et al What a man wants: understanding the challenges and motivations to physical activity participation and healthy eating in middle-aged Australian men. Am J Mens Health. 2012;6(6):453–61. doi: 10.1177/1557988312444718 22516565 10.1177/1557988312444718

[32] Sullivan AN, Lachman ME. Behavior change with fitness technology in sedentary adults: a review of the evidence for increasing physical cctivity. Front Public Health. 2016;4:289 doi: 10.3389/fpubh.2016.00289 28123997 10.3389/fpubh.2016.00289 PMC5225122

[33] Mansi S, Milosavljevic S, Tumilty S, Hendrick P, Higgs C, Baxter DG. Investigating the effect of a 3-month workplace-based pedometer-driven walking programme on health-related quality of life in meat processing workers: a feasibility study within a randomized controlled trial. BMC Pub Health. 2015;15:410. 25895747 10.1186/s12889-015-1736-z PMC4431031

[34] Puig-Ribera A, Bort-Roig J, Gonzalez-Suarez AM, Martinez-Lemos I, Gine-Garriga M, Fortuno J, et al Patterns of impact resulting from a 'sit less, move more' web-based program in sedentary office employees. PLoS One. 2015;10(4):e0122474 doi: 10.1371/journal.pone.0122474 25830782 10.1371/journal.pone.0122474 PMC4382156

[35] Sanders JP, Loveday A, Pearson N, Edwardson C, Yates T, Biddle SJH, et al Devices for self-monitoring sedentary time or physical activity: a scoping review. J Med Internet Res. 2016;18(5):e90 doi: 10.2196/jmir.5373 27145905 10.2196/jmir.5373 PMC4871995

[36] Trost SG, Owen N, Bauman AE, Sallis JF, Brown W. Correlates of adults' participation in physical activity: review and update. Med Sci Sports Exerc. 2002;34(12):1996–2001. doi: 10.1249/01.MSS.0000038974.76900.92 12471307 10.1097/00005768-200212000-00020

[37] Trivedi T, Liu J, Probst J, Merchant A, Jones S, A. M. Obesity and obesity-related behaviors among rural and urban adults in the USA. Rural Remote Health. 2015;15:3267. 26458564

[38] Brown WJ, Mummery K, Eakin E, Schofield G. 10,000 Steps Rockhampton: evaluation of a whole community approach to improving population levels of physical activity. J Phys Act Health. 2006;3(1):1.

[39] Mummery WK, Schofield G, Hinchliffe A, Joyner K, Brown W. Dissemination of a community-based physical activity project: the case of 10,000 steps. J Sci Med Sport. 2006;9(5):424–30. doi: 10.1016/j.jsams.2006.06.015 16890489 10.1016/j.jsams.2006.06.015

[40] Jennings CA, Berry TR, Carson V, Culos-Reed N, Duncan MJ, Loitz CC, et al UWALK: the development of a multi-strategy, community-wide physical activity program. Transl Behav Med. 2016;7(1):16–27. 10.1007/s13142-016-0417-5 PMC5352639 27282432

[41] Flores Mateo G, Granado-Font E, Ferre-Grau C, Montana-Carreras X. Mobile Phone Apps to Promote Weight Loss and Increase Physical Activity: A Systematic Review and Meta-Analysis. J Med Internet Res. 2015;17(11):e253 doi: 10.2196/jmir.4836 26554314 10.2196/jmir.4836 PMC4704965

[42] Goode AP, Hall KS, Batch BC, Huffman KM, Hastings SN, Allen KD, et al The Impact of Interventions that Integrate Accelerometers on Physical Activity and Weight Loss: A Systematic Review. Ann Behav Med. 2016:1–15. doi: 10.1007/s12160-015-9725-0 27565168 10.1007/s12160-016-9829-1 PMC5253097

[43] Higgins JP. Smartphone applications for patients' health and fitness. Am J Med. 2016;129(1):11–9. doi: 10.1016/j.amjmed.2015.05.038 26091764 10.1016/j.amjmed.2015.05.038

[44] Dempsey W, Liao P, Klasnja P, Nahum-Shani I, Murphy SA. Randomised trials for the Fitbit generation. Signif (Oxf). 2015;12(6):20–3. 26807137 10.1111/j.1740-9713.2015.00863.x PMC4721268

