# Supplementary material for: Consumer physical activity tracking device ownership and use among a population-based sample of adults
Source: PLoS One. 2018 Jan 2;13(1):e0189298. doi: 10.1371/journal.pone.0189298 (PMC5749689; doi:10.1371/journal.pone.0189298)
Supplement: S1 Table — (DOCX) [file pone.0189298.s001.docx]

**Supplement 1**

**Table. Number of completions by call attempts**

| **Call Attempts** | **# of Completes** | **Valid Percent** | **Cumulative Percent** |
| --- | --- | --- | --- |
| 1 | 579 | 47.7 | 47.7 |
| 2 | 362 | 29.8 | 77.4 |
| 3 | 154 | 12.7 | 90.1 |
| 4 | 70 | 5.8 | 95.9 |
| 5 | 25 | 2.1 | 97.9 |
| 6 | 18 | 1.5 | 99.4 |
| Over 6 attempts* | 7 | 0.6 | 100.0 |
| **Total** | 1,215 | 100.0 |  |

*Over 6 attempts were made in cases in which additional callbacks were requested by respondents on the 5^th^ attempt.
